# Chemically Defined Formulas, Symbiotics and Cow’s Milk Protein Allergy

**DOI:** 10.3390/nu14020299

**Published:** 2022-01-11

**Authors:** Jean-Pascal De Bandt

**Affiliations:** 1 Service de Biochimie, Hôpital Cochin, APHP.Centre—Université de Paris, 75014 Paris, France; jean-pascal.de-bandt@u-paris.fr; 2 INSERM UMR-S 1124, Université de Paris, 75006 Paris, France

**Keywords:** probiotics, prebiotics, intestine, dysbiosis, infection

Cow’s milk protein (CMP) allergy (CMPA) is the earliest and most common food allergy in children. Its management is based on the avoidance of intact cow’s milk proteins; it can conveniently be based on extensively hydrolyzed proteins (EHP). However, it is generally considered that nearly 10% of children with CMPA will not tolerate EHP and will require amino acid formulas (AAF) [1].

In the broader context of nutritional assistance, the first nutritional formulas developed were the so-called space diets, whose aim was to limit digestive residues in the context of space missions [2] and consisted of mixtures of amino acids for protein support. In the field of enteral nutrition (EN), they were known as elemental diets. Apart from cost and technical problems (limited solubility and/or stability of some amino acids), these mixtures are moderately tolerated (owing to perturbations of digestive tract motricity linked to high osmolality). They have progressively been replaced, whenever possible, by semi-elemental (partially hydrolyzed) and thereafter polymeric (non-hydrolyzed) diets; polymeric diets have been the most widely evaluated and have demonstrated their efficacy in the most common situations of EN, which is not the case for elemental and semi-elemental diets. In the context of CMPA, given that the most common source of protein in enteral formulas is cow’s milk and that the degree of hydrolysis of semi-elemental diets is insufficient to eliminate cow’s milk allergens, only elemental diets are indicated for a significant number of children.

This brings us back to the original question: is there a particular issue associated with the oral/enteral use of chemically defined diets and, more specifically, diets in which nitrogen is provided in the form of free amino acids?

Indeed, in terms of nitrogen supply, nitrogen absorption, balance and efficiency are notably influenced by proteins’ molecular form, i.e., intact, partially or completely hydrolyzed [3,4]. Protein absorption has long been considered to depend on peptide terminal hydrolysis, as is the case for carbohydrates, at the brush border of enterocytes for uptake as free amino acids via various amino acid transporters. Moreover, the intestine is characterized by a high mucosal renewal rate, meaning that every event susceptible to alter this renewal (malnutrition, inflammation, etc.) will alter mucosal trophicity and amino acid uptake. In the early 1990s, it was shown that small peptides can be taken by the enterocytes via a new transporter called PepT1 [5], with the amino acids being transferred to the blood after intracellular hydrolysis. Furthermore, it was suggested that Pept1 can effectively rescue nitrogen absorption in inflammatory situations [6]. It was then hypothesized that providing peptides could thus improve refeeding efficiency through active uptake by this peptide transporter [7]. Currently, the SLC15 oligopeptide transporter family appears to be responsible for the majority of protein absorption [8]. However, it is known that free amino acids are efficiently taken up by the enterocyte, their major drawback being hyperosmolarity, which can limit tolerance (evident in general for boluses greater than 10 g for many amino acids in their free form). The important question remains that of nutritional efficiency. On this point, unfortunately, the few data available concern mainly the comparison between whole proteins and protein hydrolysates. Improved nitrogen homeostasis with protein hydrolysates compared to whole proteins has been shown in adults [9,10,11]. Conversely, experiments in rodents have suggested that fully hydrolyzed proteins may have negative effects [12]. In a model of acute malnutrition-refeeding in young rats, we observed that peptides, partially hydrolyzed proteins and whole proteins were all effective in the very short term, but partially hydrolyzed protein appeared to be more effective in the medium term in restoring lean body mass [13]. Interestingly, in the RCT by Burks et al. [14], growth and tolerance were similar in healthy term infants receiving AAF or EHF from 14 to 120 days of age. However, in its 2010 guidelines [1], the World allergy organization concluded, after an extensive review of the literature, that the demonstration of benefit of AAF over EHF was uncertain.

A possible explanation for these mixed conclusions could be an inherent flaw in all these preparations: a composition based solely on macro- and micronutrient supply and that does not take into account the importance of non-nutritive compounds normally present in our diet for our digestive functionality. Although interest in probiotic bacteria dates back to the 1950s, it has gradually become apparent that certain bacteria of the genera Bifidobacterium and Lactobacillus can have beneficial effects on health (in particular with regard to allergic symptoms, such as atopy, or digestive disorders, such as traveler’s diarrhea) via their effect on digestive trophicity. One of the difficulties in using these probiotics is the limited persistence of these microorganisms in the intestine. It therefore appears necessary to provide, along with the probiotics, elements that will promote their growth, the prebiotics, hence the concept of symbiotics that simultaneously provide pre- and probiotics [15]. This question has received renewed interest with the work of Gordon’s team [16], showing the close relationship between intestinal microbiota and metabolism. Since then, many studies have been devoted to the study of the intestinal microbiota and have highlighted numerous situations of microbiota imbalance (dysbiosis) in the course of different pathologies. This has paved the way for the study of interactions between symbiotics and microbiota and their ability to correct dysbiosis. Symbiotics could therefore be of particular interest when using chemically defined formulas in which the absence of dietary fiber will promote dysbiosis, possibly already present due to the underlying pathology, and weaken the intestinal barrier [17].

This has prompted several studies on prebiotic supplementation in CMPA. A 2019 meta-analysis by Qamer et al. [18] retrieved 10 randomized control trials (RCTs; *n* = 422 patients with probiotics and 423 control) with hematochezia and acquisition of tolerance as primary endpoints. These authors concluded to a higher acquisition of tolerance to CMP with probiotics but with low-quality evidence. Of note, of these 10 studies, 7 used probiotics and 3 synbiotics and, in terms of nutritional support, included breastfeeding, extensively hydrolyzed casein or whey, or AAFs depending on the studies; the heterogeneity and limited number of evaluable patients for the selected criteria probably explain the weakness of the evidence. The meta-analysis of Sorensen et al. [19] goes a step further in demonstrating the value of symbiotics in the management of CMPA by addressing the most problematic aspect of this management, namely the provision of AAFs. This work identified four RCTs comparing AAFs without and with symbiotics for a population of 410 children with CMPA with a mean intervention duration of approximately 27 weeks. Like Qamer et al. [18], Sorensen et al. [19] concluded that the efficacy of treatment was similar in terms of symptom progression and growth. More importantly, these authors also considered infections, medications and changes in fecal microbiota as criteria. They show a significant reduction in the prevalence of infections [Odd Ratio (OR) 0.35 (95% CI 0.19 to 0.67), *p* = 0.001] in children receiving symbiotics. In the pooled analysis, the weighted average percentage of infants receiving anti-infective treatment was 15% with symbiotics vs. 33% without. Analysis of the fecal microbiota showed that symbiotic administration was associated with a significant increase in the percentage of bifidobacterium bacteria (difference in means 31.75, 95% CI 26.04–37.45, *p* < 0.0001) and a significant decrease in the percentages of Eubacterium rectale and Clostridium coccoides bacteria (difference in means −19.06, 95% CI −23.15 to −14.97, *p* < 0.0001). CMPA is associated with dysbiosis, and these data suggest a significant improvement that may promote intestinal barrier homeostasis. It is therefore tempting to speculate that the reduction in infectious complications was a direct result of an improvement in gut trophicity mediated by symbiotics. Finally, the results of the meta-analysis by Sorensen et al. [19], while encouraging the continuation of RCTs on the subject in order to base these conclusions on larger numbers, draw attention to the need for longer-term evaluation and a focus on certain points that are often inadequately evaluated in the overall management of children, such as quality of life or total costs.
